# Tuning Alginate Bioink Stiffness and Composition for Controlled Growth Factor Delivery and to Spatially Direct MSC Fate within Bioprinted Tissues

**DOI:** 10.1038/s41598-017-17286-1

**Published:** 2017-12-06

**Authors:** Fiona E. Freeman, Daniel J. Kelly

**Affiliations:** 1 Trinity Centre for Bioengineering, Trinity Biomedical Sciences Institute, Trinity College Dublin, Dublin, Ireland; 2 Department of Mechanical and Manufacturing Engineering, School of Engineering, Trinity College Dublin, Dublin, Ireland; 3 Department of Anatomy, Royal College of Surgeons in Ireland, Dublin, Ireland; 4 Advanced Materials and Bioengineering Research Centre (AMBER), Royal College of Surgeons in Ireland and Trinity College Dublin, Dublin, Ireland

## Abstract

Alginate is a commonly used bioink in 3D bioprinting. Matrix stiffness is a key determinant of mesenchymal stem cell (MSC) differentiation, suggesting that modulation of alginate bioink mechanical properties represents a promising strategy to spatially regulate MSC fate within bioprinted tissues. In this study, we define a printability window for alginate of differing molecular weight (MW) by systematically varying the ratio of alginate to ionic crosslinker within the bioink. We demonstrate that the MW of such alginate bioinks, as well as the choice of ionic crosslinker, can be tuned to control the mechanical properties (Young’s Modulus, Degradation Rate) of 3D printed constructs. These same factors are also shown to influence growth factor release from the bioinks. We next explored if spatially modulating the stiffness of 3D bioprinted hydrogels could be used to direct MSC fate inside printed tissues. Using the same alginate and crosslinker, but varying the crosslinking ratio, it is possible to bioprint constructs with spatially varying mechanical microenvironments. Moreover, these spatially varying microenvironments were found to have a significant effect on the fate of MSCs within the alginate bioinks, with stiffer regions of the bioprinted construct preferentially supporting osteogenesis over adipogenesis.

## Introduction

Tissue engineering (TE) is a promising strategy for replacing, repairing or regenerating damaged tissues and organs. TE strategies typically incorporate cells, biomaterials and signals (e.g. growth factors), with the goal of developing a construct that once implanted will promote tissue regeneration. A limitation of current TE strategies is their relatively poor spatial control of the distribution of cells, matrix components and bioactive cues within the engineered construct^1^. One way of overcoming this limitation is through the use of emerging additive biomanufacturing strategies^1,2^. In particular, 3D bioprinting^3,4^ allows for the development of complex anatomically accurate scaffold geometries that also mimic aspects of the composition and organisation of native tissues through the simultaneous deposition of biomaterials, cells, proteins and/or genes in defined locations^1,5,6^.

One of the main challenges with bioprinting cell laden constructs is the identification of an appropriate bioink, as the material not only needs to have the necessary structural and mechanical properties, but should also protect the cells from damage during printing and ultimately provide them with an appropriate environment to direct or control their phenotype and function. One of the most common natural materials used for hydrogel based tissue engineering and drug delivery is alginate^7–9^. It is a highly biocompatible hydrogel whose physical properties can potentially be tailored to direct 3D cell growth and differentiation both *in vitro* and *in vivo* ^10–12^. Moreover, alginate is characterised by a wide pore size distribution (5–200 mm) which facilitates the diffusion of large molecules in and out of the gel^13^. Alginate is also commonly used for drug/growth factor delivery, where the degradation rate of the alginate can be tuned by altering the MW of the alginate^14,15^, which can in turn vary the release rate of the drug/growth factor encapsulated. Gelation of alginate is typically induced by cations such as Ca+, making it an attractive choice as a bioink for 3D bioprinting^7–9,16–20^. At present the most frequent choice of ionic crosslinker is Calcium Chloride (CaCl_2_) as it typically leads to rapid gelation due to its high solubility in aqueous solutions^21^. Calcium sulphate (CaSO_4_) and calcium carbonate (CaCO_3_) are the other two popular choices of crosslinkers, however due to their lower solubility within an aqueous solution the gelation time is slower. The choice of ionic crosslinker can also have a significant effect on the mechanical integrity of an alginate hydrogel-based bioink^21,22^.

One of the potential limitations associated with the use of alginate for *in vivo* tissue regeneration is that it is in general non-degradable by mammals, as they lack the enzymes needed to break down the polymer chains^21^. One application where degradability is vital is in bone tissue engineering, as residual biomaterial can impede vascularization and bone formation^23–26^. The degradation rate and mechanical properties of alginate gels can be varied by adjusting its MW^14,15,22,27–29^. One of the most common methods of reducing the MW of the alginate is through γ-irradiation. To date, the majority of *in vivo* bone tissue engineering approaches that utilise alginate as a hydrogel use a γ-irradiated version of this biomaterial as it has significantly faster degradation^14^, which correlates with increased bone formation within segmental defects^24,25,30–34^. In the context of bioprinting with alginate, decreasing its MW reduces its viscosity, making it difficult to print with. Therefore, there is likely a trade-off to ensure the MW of an alginate is low enough to degrade *in vivo* but high enough to have a viscosity suitable for printing.

*In vivo* cells have also been shown to adhere, contract and migrate through an array of tissues varying from soft brain tissue to the stiff osteoid of remodelling bone^35^. The mechanical properties of the extracellular matrix influence focal adhesion structure and the cytoskeleton of differentiating cells^36–41^. Furthermore, the differentiation pathway of MSCs is strongly influenced by the rigidity of the underlying substrate^35,42,43^. MSCs cultured on soft collagen coated gels exhibit spindle-like characteristics of primary neurons, whereas on stiffer matrices MSCs have been shown to adopt a cuboidal osteoblast morphology^35^. Matrix stiffness has also been shown to regulate MSC differentiation in 3D alginate gels, with adipogenesis supported in soft alginate hydrogels (Young’s Modulus of 2.5-5kPa) and osteogenesis supported in stiffer hydrogels (Young’s Modulus of 11–30 kPa)^43^. This suggests that by modulating the stiffness of an alginate bioink we might be able to regulate MSC fate within a 3D bioprinted construct.

The MW of alginate, choice of crosslinker and gelling conditions have all been shown to have a significant effect on the physical properties of alginate. Furthermore, it has been demonstrated that alginate stiffness is a key determinant of MSC differentiation. It is unclear, however, how these factors impact the printability of alginate, cell viability post-printing and ultimately the phenotype of progenitor cells embedded within 3D bioprinted constructs. The four main objectives of this study were: (1) to investigate the effect of MW, gelling conditions and choice of crosslinker on the bioprintability and mechanical properties of alginate bioinks, (2) to determine how such bioprinting conditions regulate MSC viability post-printing, (3) to examine how the rate of growth factor release from alginate bioinks is influenced by its MW, and (4) to demonstrate that spatially modulating the stiffness of 3D bioprinted constructs can be used to regulate MSC fate and hence engineer complex tissues. We compared the printability, mechanical properties and post-printing cell viability associated with two alginate-based hydrogels of varying molecular weights using a range printing conditions. We then investigated how these parameters can regulate MSC fate within a spatially defined 3D bioprinted construct.

## Results & Discussion

### Comparison of Bioink Printability

**Figure 1:**
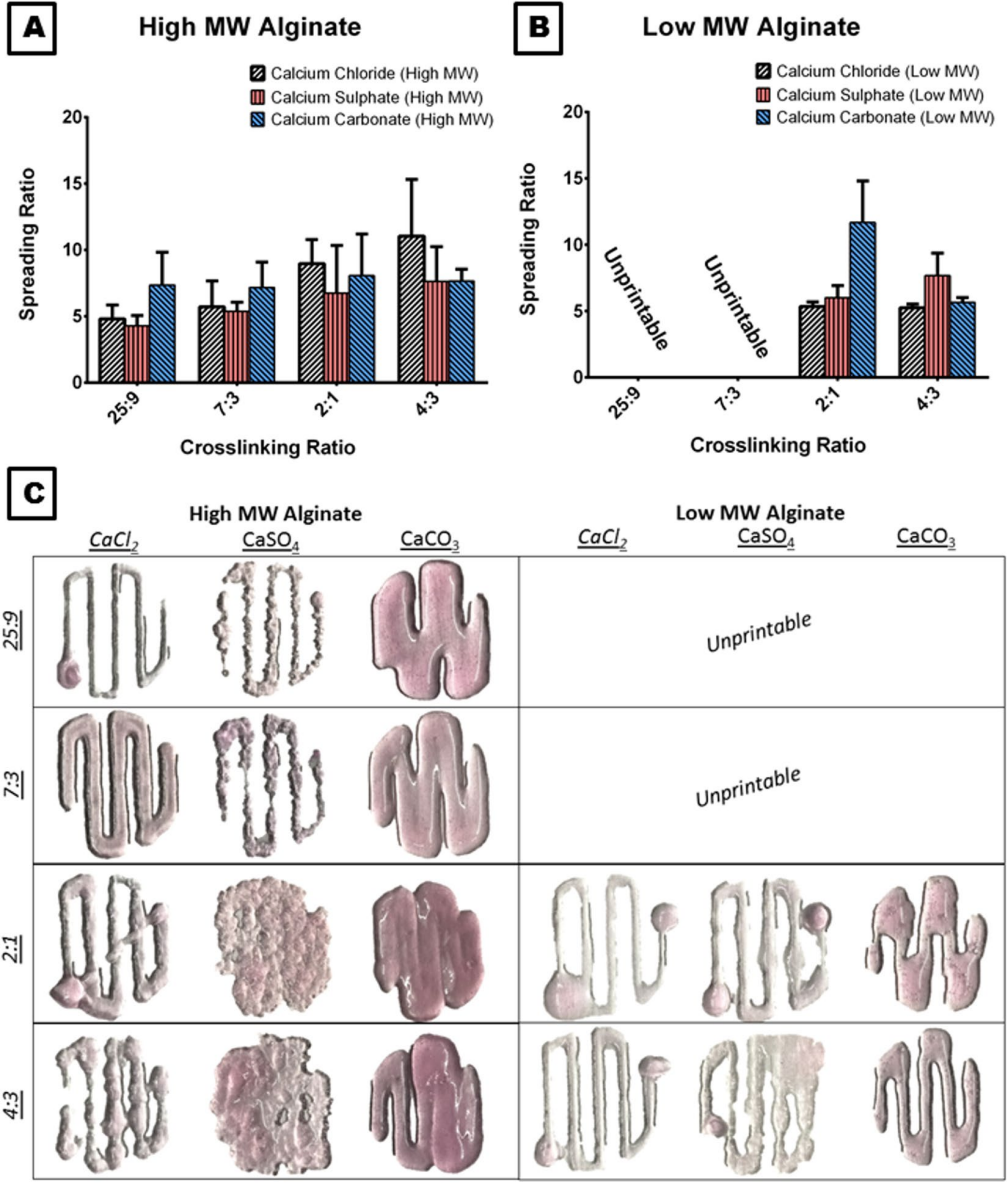
Evaluation of the printability of the bioinks. Spreading Ratio for **(A)** high MW and **(B)** low MW Alginate at 25:9, 7:3, 2:1 and 4:3 crosslinking ratios (alginate: crosslinker) for CaCl_2_, CaSO_4_ and CaCO_3_ crosslinkers. Error bars denote standard deviation, n = 6. **(C)** Representative images of the design pattern used to determine spreading ratios at all of the crosslinking ratios for all of the crosslinkers. All bioinks were prepared in DMEM.

To establish the optimum printing parameters, the printability of the two MW alginates (28,000 or 75,000 g/mol) was evaluated at a variety of crosslinking ratios (using bioinks that were prepared in high glucose DMEM). The width of the printed filament was measured using ImageJ software and divided by the internal needle diameter, to obtain the spreading ratio. Lower spreading ratios are desirable to allow the fabrication of cell laden hydrogel structures with high precision. The lowest spreading ratio for the high MW alginate was observed at a crosslinking ratio of 25:9 (alginate: crosslinker) regardless of the choice of crosslinker, see Fig. 1(A). At the 25:9 crosslinking ratio, using CaSO_4_ as a crosslinker resulted in the lowest spreading ratio of 4.3 ± 0.77. At any lower crosslinking ratios, the bioink became over crosslinked and made it very difficult to recapitulate a prescribed printing pattern as seen in Fig. 1(C). In contrast, the low MW alginate bioink had the lowest spreading ratio at a crosslinking ratio of 4:3 regardless of crosslinker. At this ratio, it was the CaCl_2_ crosslinker that resulted in the lowest spreading ratio of 5.3 ± 0.28, see Fig. 1(B). As the viscosity of the low MW alginate was so low, it was completely unprintable at crosslinking ratios of 25:9 and 7:3 regardless of the choice of crosslinker. These results demonstrate that the molecular weight of alginate bioinks has a significant effect on its printability, and that the amount of crosslinker required to generate a printable hydrogel is 2.5 times greater when using a low MW alginate compared to high MW alginate. Hence the optimum crosslinking ratio for high and low MW alginates was 25:9 and 4:3 respectively. These crosslinking ratios are the pre-determined optimum crosslinking ratios for each ink used hereafter in this study.

It has been previously established^44^ that the mechanical properties of alginate depend on phosphate concentrations. Therefore, preparing an alginate solution in PBS over DMEM can significantly affect both the mechanical properties and hence printability of an alginate bioink. It was found that preparing a high MW alginate solution in PBS over DMEM at the same crosslinking ratio (25:9) significantly increased the spreading ratio if CaCO_3_ was used as the crosslinker, see Supplementary Fig. 1A. Similarly, preparing low MW alginate in PBS over DMEM at the same crosslinking ratio (4:3) significantly increased the spreading ratio if CaCl_2_ is used as the crosslinker, see Supplementary Fig. 1B. Therefore, not only does the MW of alginate and choice of ionic crosslinker influence the printability of the bioink, but also the choice of gelling conditions (PBS versus DMEM).

### Evaluation of Cell Viability within Bioinks

**Figure 2:**
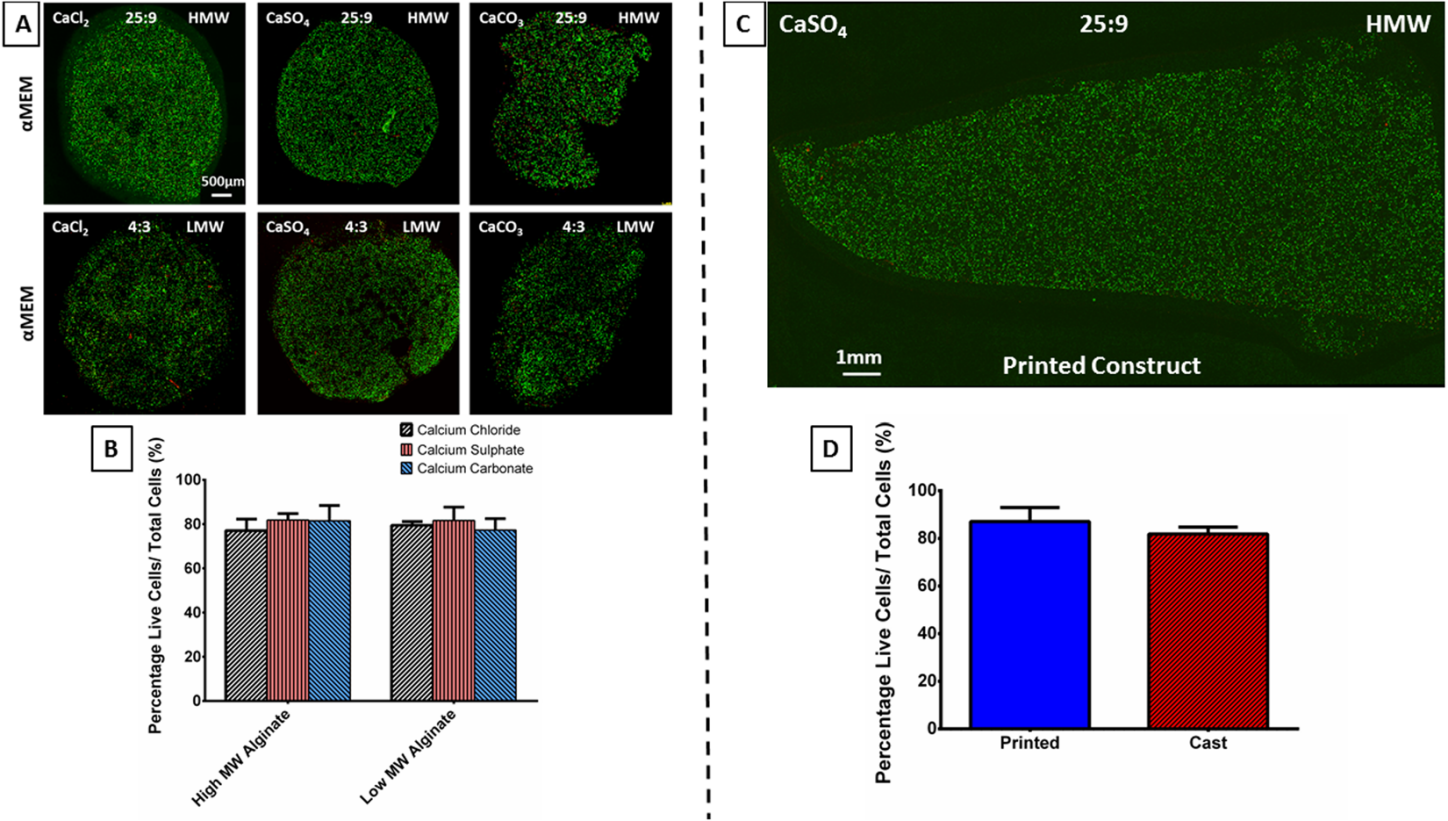
Cell viability of the bioinks. **(A)** Representative images of Live/Dead staining used to determine cell viability of CaCl_2_, CaSO_4_ and CaCO_3_ crosslinkers for both high (HMW) and low MW (LMW) **cast** alginate constructs at the previously determined optimum crosslinking ratio (25:9 and 4:3 respectively). **(B)** Quantitative analysis of the cell viability for all of the bioinks. All bioinks were prepared in DMEM. Error bars denote standard deviation, *n = *5. **(C)** Representative image of Live/Dead staining of a **3D printed** construct printed with HMW alginate crosslinked with CaSO4 at the optimum crosslinking ratio of 25:9. **(D)** Quantitative analysis of the cell viability for **cast** and **3D printed** high MW alginate crosslinked with CaSO_4_. All bioinks were prepared in DMEM. Error bars denote standard deviation, n = 5.

During the printing procedure, there are numerous factors that can affect cell viability. These include gelling conditions, fabrication time and the shear stress the cells experience during the crosslinking and printing procedures^1^, as well as any post-printing crosslinking. Therefore, these factors must be considered when determining the viability of the bioprinting process. We first sought to evaluate the chemical cytocompatibility of the three crosslinkers in isolation from other printing factors. To this end, bioinks were prepared exactly as if they would be prepared for printing but were then cast into an agarose mould to create small constructs. All bioinks regardless of the choice of alginate or crosslinker supported high levels of cell viability, with no differences between the groups, see Fig. 2(A). The bioinks prepared in PBS also showed no loss of cell viability, see Supplementary Fig. 1G. This was confirmed by quantitative analysis showing that after 24 hours all groups had at least 70% cell viability, see Fig. 2(B). We next assessed if the additional shear stress associated with the actual printing process would negatively affect cell viability. Given its higher viscosity, a high MW alginate was prepared in growth medium and crosslinked with CaSO_4_, before printing using a conical needle which has previously been shown to reduce the levels of shear stress cells are exposed to during printing^45^. There was high cell viability (85.02% ± 5.94) present within all of the constructs 24 hours after printing see Fig. 2(C), similar to those previously reported^8,45–47^ and comparable to that observed in cast hydrogels Fig. 2(D). Therefore, the printing conditions evaluated here (gelling conditions, fabrication time and shear stress the cells experience during the crosslinking and printing procedure) have only a small effect on cell viability within 3D printed alginate constructs.

### Mechanical Properties of the Bioinks Post-Printing

**Figure 3:**
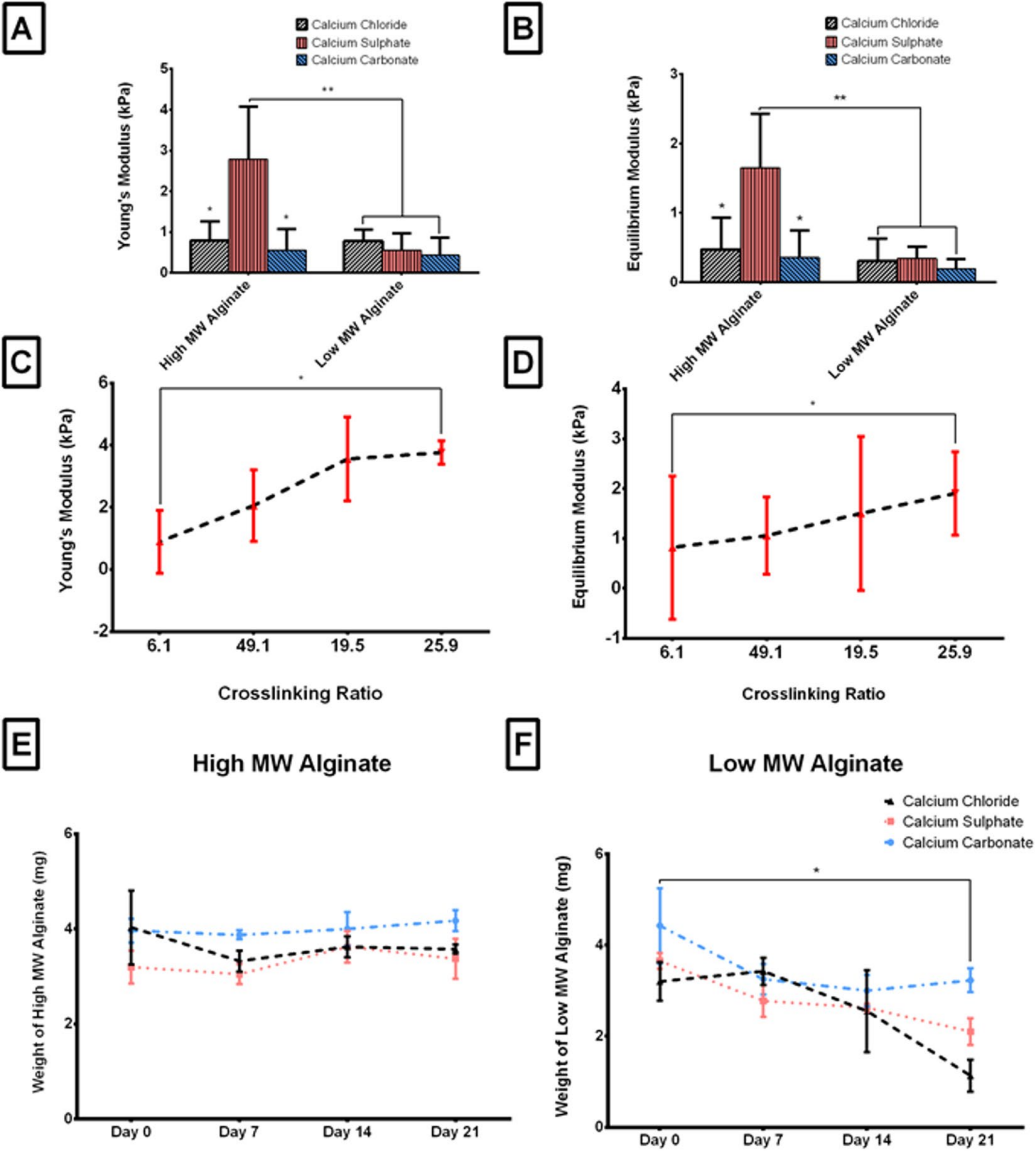
Mechanical Properties of the bioinks. **(A)** Young’s Modulus and **(B)** Equilibrium Modulus for the high and low MW **3D printed** alginate crosslinked with all three crosslinkers at the previously determined optimum crosslinking ratio (25:9 and 4:3 respectively). *p < 0.05 and **p < 0.001 vs. high MW CaSO_4_ bioink. **(C)** Young’s Modulus and **(D)** Equilibrium Modulus for the high MW **3D printed** Alginate crosslinked with CaSO_4_ at a variety of crosslinking ratios (alginate:crosslinker; 6:1, 49:10, 19:5 and 25:9). *p < 0.05 vs. 6:1 crosslinking ratio. Degradation rate of **(E)** high and **(F)** low MW **cast** alginate. *p < 0.05 vs. weight at Day 0. All bioinks were prepared in DMEM. Error bars denote standard deviation, n = 6.

As previously stated, the molecular weight of alginate has a significant effect on the mechanical properties of an alginate hydrogel^14,15,22,27–29^. In agreement with this literature, the mechanical properties of the printed constructs increased with increasing alginate MW, see Fig. 3(A,B). Interestingly, the choice of ionic crosslinker also had a significant effect on the mechanical properties of the constructs. Specifically, the Young’s Modulus and Equilibrium Modulus was significantly higher in the high MW alginate bioinks crosslinked with CaSO_4_ compared to the same alginate crosslinked with CaCO_3_ and CaCl_2_. This change in mechanical properties may be because the CaCl_2_ has a high solubility in aqueous solutions, which leads to rapid gelation that can reduce construct mechanical properties. Previous studies have shown that a rapid gelation time leads to non-uniform crosslinking, which in turn can negatively impact mechanical properties^21,22^. In contrast, CaSO_4_ and CaCO_3_ have lower solubility within an aqueous solution, resulting in slower more uniform gelation that can improve the mechanical properties of the resulting hydrogel. A limitation of calcium carbonate is that it is not soluble in water at a neutral pH, therefore to dissociate the Ca^2+^ ions from the CaCO_3_ Glucono-δ-lactone needs to be added to lower the pH^21,22^. This lowering of the pH may be negatively impacting the mechanical properties of the bioink.

The Young’s Modulus of an alginate bioink can also be tuned through varying the crosslinking ratio (alginate: crosslinker) alone, with stiffness increasing with relative increases in available crosslinker, see Fig. 3(C,D). Furthermore, previous studies have also shown that preparing the alginate solution in PBS over DMEM can also significantly reduce the mechanical properties of the hydrogels, as phosphate transiently binds to Ca^+^ ions to cause slower gelling and a reduction of mechanical integrity^27,44^. This reduction in mechanical properties was only seen in the high MW alginates crosslinked with CaSO_4_, otherwise there was little difference in mechanical properties between the bioinks prepared in PBS versus DMEM, see Supplementary Fig. 1(C,D). Thus, the choice of ionic crosslinker has a significant effect on the mechanical properties of high MW alginates prepared in either DMEM or PBS. As there was no significant difference in cell viability between PBS and αMEM, and given that the mechanical properties and spreading ratio (see section 2.1) were significantly better in the bioinks generated in αMEM, it was chosen as main bioink preparation method hereafter in this study. It should be noted that DMEM does contain 0.02 mM of CaCl_2_, however this is such a low concentration of CaCl_2_ that it should have negligible effect on the crosslinking potential of the bioink. Moreover, all the groups were prepared in the same DMEM so the differences seen between the groups are not due to the extra presence of CaCl_2_.

Degradability is another critical mechanical property of a hydrogel, specifically those used for tissue engineering approaches, with for example faster degradation^14^ associated with increased bone formation^24,25,30–34^. To increase its degradability, previous studies have manipulated the MW weights of alginates, with higher molecular weights associated with slower degradation rates^21^. As expected, in this study the high MW alginate showed little to no degradation over the 21 days in culture regardless of the choice of crosslinker, see Fig. 3(E). In contrast, the low MW alginate underwent dramatic degradation from Day 0 to Day 21, regardless of choice of crosslinker, see Fig. 3(F). Interestingly, the low MW alginate bioinks crosslinked with CaSO_4_ and CaCl_2_ degraded significantly faster than those crosslinked with CaCO_3_. Therefore lowering the pH of the solution can significantly affect both the young’s modulus and degradability of the resulting bioink. Taken together, the choice of ionic crosslinker has a significant effect on the degradability but only when using a low molecular weight alginate bioink.

### Growth Factor Retention and Release within Alginate Bioinks of varying molecular weight

**Figure 4:**
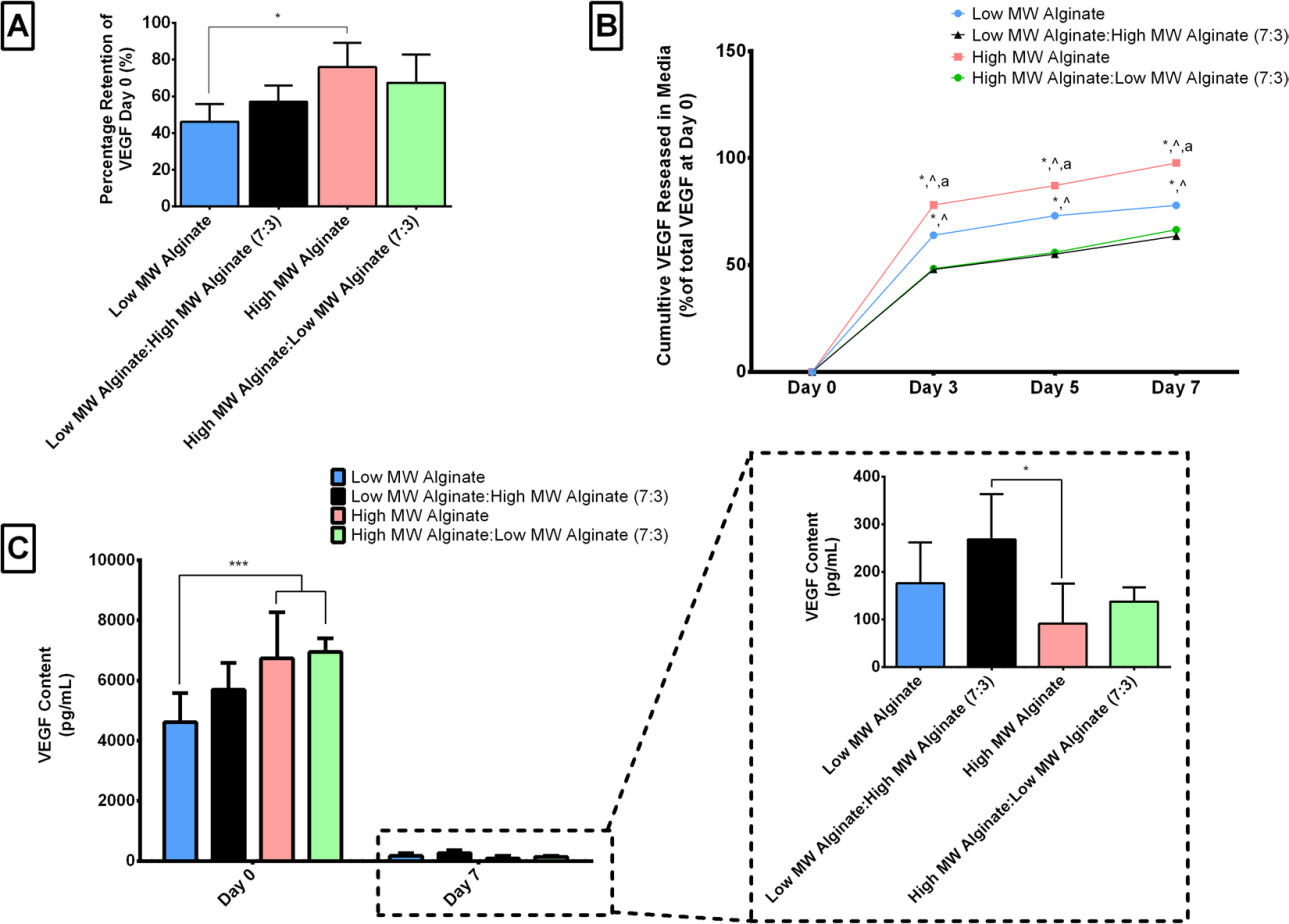
VEGF Retention and Release from the Alginate bioinks. **(A)** Percentage of VEGF present within the alginate bioinks immediately post printing. *p < 0.05 **(B)** Cumulative VEGF Release over 7 days in culture. *p < 0.05 vs. low MW Alginate: high MW Alginate (7:3), ^p < 0.05 vs. high MW Alginate: low MW Alginate, and ^a^p < 0.05 vs. low MW Alginate. **(C)** VEGF Content within the Alginate Bioinks at Day 0 and Day 7 *p < 0.05 and ***p < 0.001. Error bars denote standard deviation, n = 5.

In general, the release rate of proteins from an alginate gel is relatively rapid due to the high porosity and hydrophilic nature of the gels^21,48,49^. Similar results were observed in this study, as straight after printing process only 45–75% of VEGF initially loaded into the alginate was present in the printed construct, see Fig. 4(A). As might be expected based on the results of previous studies using alginate to control growth factor release^21,48,49^, we found that the MW of the alginate also had a significant effect on the percentage of VEGF present post printing. Lowering the MW of the alginate significantly reduced (p < 0.05) VEGF retention within the bioink post-printing. Interestingly, the alginate MW not only influenced VEGF retention post-printing, but also its release from the bioink over time, see Fig. 4(B,C). The bioinks with the different blends of MW alginates released VEGF at significantly slower (p < 0.001) rates over time compared to the high and low MW alginates alone. The high MW alginate not only retained the highest amount of VEGF post printing but also released the VEGF at the fastest rate. Together, the results of this study show that when using an alginate bioink as a growth factor carrier, its MW will have a significant effect on the retention and release of the protein within the bioprinted construct. Moreover, the results show that this can also be tuned by varying the amount of high and low MW Alginate within the bioink, which agrees with previous studies using alginate as an injectable biomaterial for controlled growth factor delivery^24,30,48–50^. Future studies using alginate as a growth factor delivery vehicle should investigate the addition of other components such as hydroxyapatite^51^ or laponite^52^ as they have been shown to facilitate the adsorption and immobilization of VEGF or the use of gelatin microspheres^16,53^ to trap the growth factors within the bioprinted hydrogel and thus slow down its release rate.

### Tuning the mechanical properties of bioprinted constructs to spatially direct MSC differentiation

**Figure 5:**
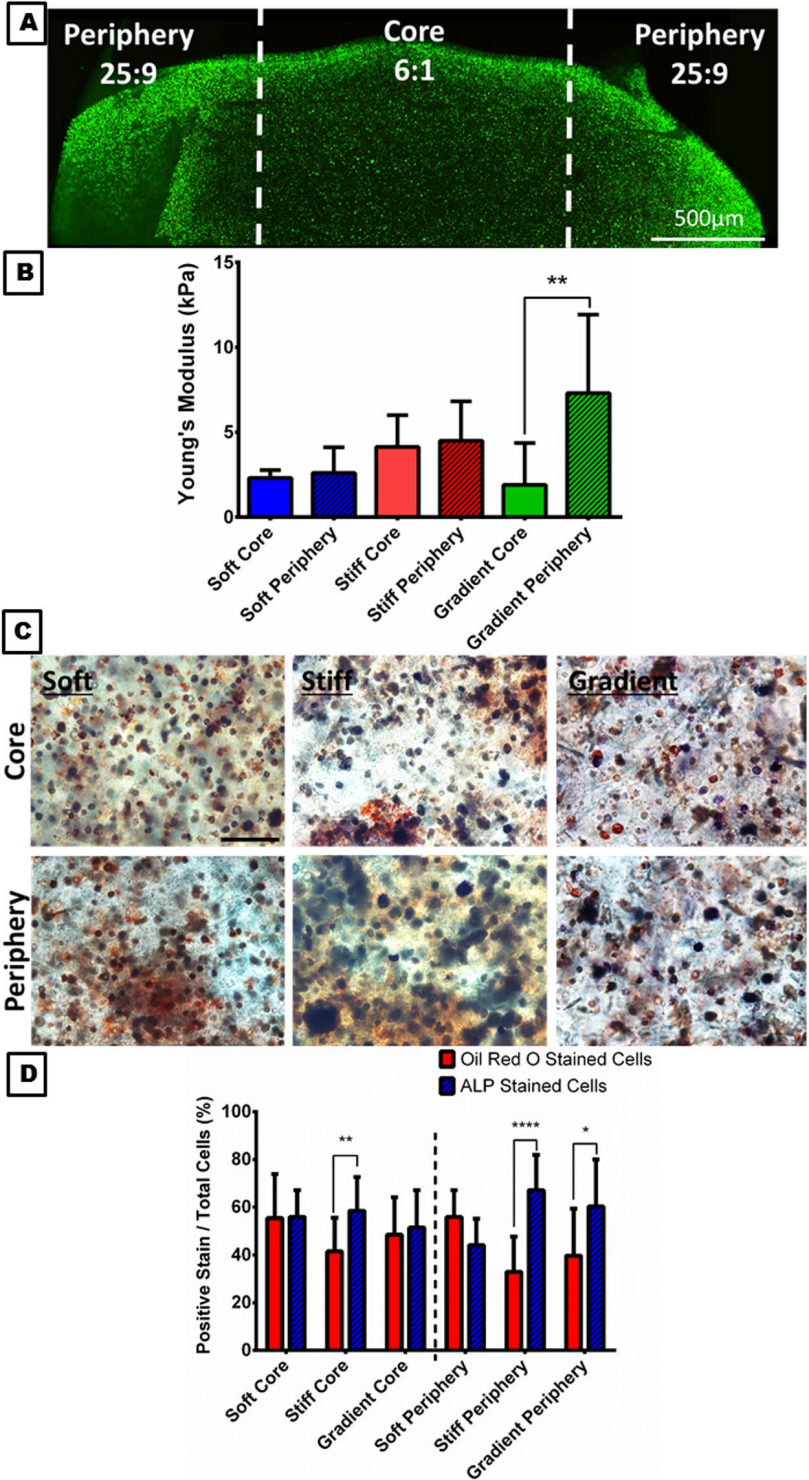
Spatially directing MSCs through varying mechanical stiffness. **(A)** Schematic of the three experimental groups with the varying stiffness. **(B)** Young’s Modulus and pre-*in vitro* culture. **p < 0.001 vs. periphery of the printed construct. **(C)** Oil Red O (red cells) and ALP (blue cells) dual staining of the core and periphery of the printed constructs 7 days in culture. **(D)** Quantitative analysis of positive Oil Red O and ALP stained cells within the core and the periphery of the three experimental groups. *p < 0.05 **p < 0.001 and ****p < 0.0001 vs. positive ALP stained cells. Error bars denote standard deviation, n = 6.

Cylindrical MSC laden constructs were bioprinted with spatially varying mechanical stiffness from the core to the periphery. There was high cell viability (87.025% ± 5.94) present within all of the constructs 24 hours after printing see Fig. 5(A), similar to those printed previously. This difference in mechanical stiffness within the printed construct was maintained during the 7 days of *in vitro* culture; see Fig. 5(B). Dual Oil Red O and ALP staining demonstrated that within the soft region of the 3D bioprinted constructs, half of the MSCs appeared to undergo osteogenesis and the other half adipogenesis, see Fig. 5(C,D). In contrast, within the stiffer region of printed constructs, significantly more MSCs preferentially underwent osteogenesis as evident by increased ALP staining. Previous studies have also demonstrated that MSCs differentiate in response to their local substrate stiffness^54–57^, and specifically that they preferentially differentiate towards an osteogenic lineage within stiffer alginate hydrogels^43^. These earlier studies exploring the role of alginate stiffness in regulating MSC differentiation incorporated adhesive ligands into the hydrogel. A potential limitation of this study is that we did not include such cell adhesive ligands into the alginate bioinks, and therefore cells may not be able to initially sense the stiffness of their surrounding environment. Mechano-sensation in these bioinks can potentially be explained by a number of different mechanisms. Shortly after hydrogel encapsulation, MSCs will deposit their own pericellular matrix (PCM) and then sense the stiffness of the hydrogel-PCM composite that surrounds them. Cellular volume changes has also been shown to play a key role in mechano-transduction within hydrogels lacking adhesive ligands^58^. These mechanisms, either alone or in combination, likely play a key role in the substrate stiffness mediated MSC differentiation observed in the printed constructs. In spite of this, the differences seen in this study might have been enhanced if cell adhesive motifs (such as RGD peptides) were incorporated into the alginate bioink. Future studies will investigate the potential of such bioinks for printing complex tissues.

The results of these studies demonstrate that by using the same alginate and crosslinker, but varying the crosslinking ratio, it is possible to bioprint constructs with spatially varying mechanical microenvironments. Moreover, by varying the printed microenvironment, MSCs can be directed towards distinct cellular lineages. One biomedical application for this is to begin to direct MSCs towards lineages seen in a long bone organ, where a cortical bone tissue surrounds a complex marrow environment supporting adipocytes and other cell populations. Future studies will investigate the co-deposition of modified alginates alongside mechanically reinforcing polymers such as PCL to engineer composite constructs with bulk mechanical properties compatible with implantation into load bearing environments, but with cell-level mechanical environments engineered to promote defined MSC differentiation pathways.

## Conclusions

When choosing, an alginate based bioink, the molecular weight of alginate, the gelling conditions and the choice of ionic crosslinker all have a significant effect on printability, mechanical properties and protein release from a 3D printed construct. Specifically, the amount of crosslinker, regardless of the choice of crosslinker, needed to generate a printable bioink is 2.5 times greater when using a low MW compared to high MW alginate. Moreover, if a high MW alginate is used CaSO_4_ produces a significantly stiffer bioink than any other ionic crosslinker investigated here. Furthermore, by using the same alginate and crosslinker, but varying the crosslinking ratio, it is possible to bioprint constructs with spatially varying mechanical microenvironments. The stiffness of these printed cellular environments can be spatially tuned to direct the differentiation of encapsulated MSCs. In conclusion, this study demonstrates how the properties of alginate bioinks can be tuned for optimal printability, controlled growth factor delivery and to spatially direct stem cell fate within bioprinted constructs. In this way, we can use 3D bioprinting to engineer spatially defined microenvironments and corresponding cellular phenotypes seen in complex solid organs such as a long bone.

## Materials and Methods

### Preparation of Bioinks

**Figure 6:**
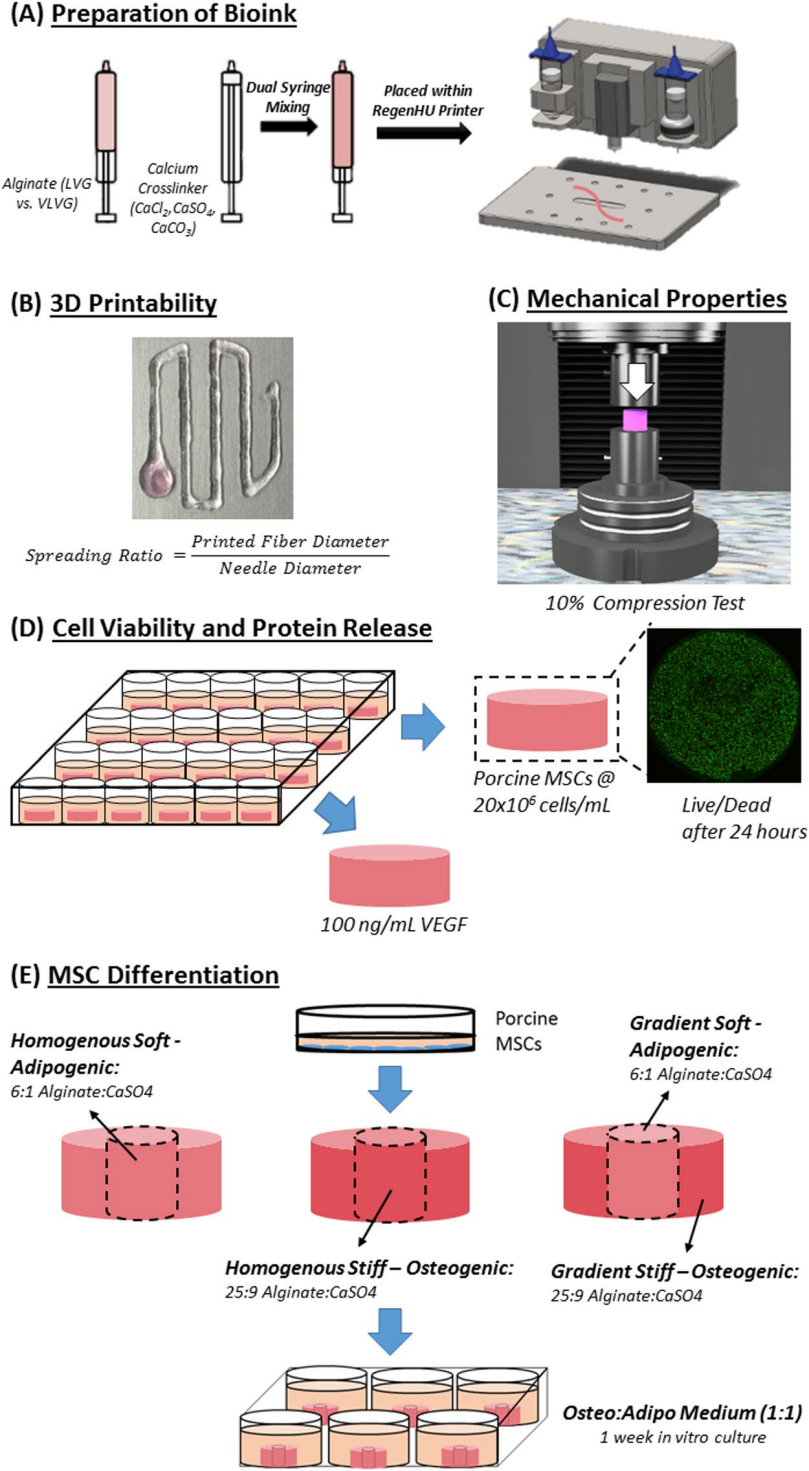
Experimental Setup. **(A)** Schematic of the preparation of the bioinks for 3D printing. **(B)** Photograph of a representative image used to determine the printability of the bioinks. **(C)** Schematic of the 10% compression test performed on the samples to determine the mechanical properties of each bioink. **(D)** Schematic of the experimental setup used to determine the cell viability of each ionic crosslinker and a representative Live/Dead image showing the cell viability of the printed constructs. **(E)** Schematic of the experimental setup used to spatially direct MSCs within bioprinted alginate constructs.

Two sodium alginate powders of varying molecular weights (MW) were used to generate bioinks of high (LVG, Pronova, M_W_ = ~75 000 g/mol) and low (VLVG, Pronova, M_W_ = 28 000 g/mol) molecular weights. The alginate solutions were prepared by dissolving the two alginate powders in either high-glucose Dulbecco’s modified eagle medium (DMEM, GlutaMAXTM;GIBCO, Biosciences, Ireland) or sterile Phosphate Buffer Solution (PBS, Sigma Aldrich) to make up a final concentration of 3.5% (w/v). Three different ionic crosslinkers were used to crosslink the alginate bioinks; Calcium Carbonate (CaCO_3_), Calcium Chloride (CaCl_2_), and Calcium Sulphate (CaSO_4_). The 60 mM solutions were prepared by dissolving CaCl_2_ and CaSO_4_ (All Sigma Aldrich) within deionised water. For the CaCO_3_ solution a 2:1 (120 mM: 60 mM) ratio of CaCO_3_ to Glucono-δ-lactone (All Sigma Aldrich) was prepared in deionised water. The 60 mM solutions were autoclaved prior to crosslinking with the alginate solutions. To form bioinks, these solutions were pre-crosslinked by mixing the alginate solutions with either CaCO_3_, CaCl_2_, or CaSO_4_ at varying volumetric ratios (v/v) (25:9, 7:3, 2:1 & 4:3) using a dual syringe approach as previously described^46^. The bioinks could crosslink for 30 minutes prior to printing, see Fig. 6(A).

### 3D Bioprinting Process

A 3D bioplotter from RegenHU (3D Discovery) was used to evaluate the printability of the generated bioinks. All bioinks were printed using a 25 G needle, at a feed rate of 8 mm/s, fibre spacing of 4 mm, and 0/90° pattern. Other varying parameters for printing of the individual bioinks are described in Table 1 below. The printability and optimum crosslinking ratio of each of the bioinks were evaluated by measuring the spreading ratio as previously described^46^, see Fig. 6(B).

$$Spreading\,Ratio=\frac{{Printed}\,{needle}\,{diameter}}{Needle\,diameter}$$

**Table 1:** Bioprinting processing parameters for each bioink prepared in DMEM.

*High MW Alginate*												
*Crosslinker*	*CaCl*_2_				*CaSO*_4_				*CaCO*_3_			
*Crosslinking Ratio*	25:9	7:3	2:1	4:3	25:9	7:3	2:1	4:3	25:9	7:3	2:1	4:3
*Extrusion Pressure (bar)*	0.7	0.9	2	2.6	0.2	0.4	0.4	0.5	0.2	0.4	0.4	0.6
***Low MW Alginate***												
***Crosslinker***	***CaCl***_**2**_				***CaSO***_**4**_				***CaCO***_**3**_			
*Crosslinking Ratio*	25:9	7:3	2:1	4:3	25:9	7:3	2:1	4:3	25:9	7:3	2:1	4:3
*Extrusion Pressure (bar)*	NA	NA	0.1	0.1	NA	NA	0.1	0.1	NA	NA	0.1	0.1

### Mechanical Testing

High and low MW alginate solutions of 3.5% (w/v) were prepared in either PBS or high glucose DMEM, as described in Section 4.1, and pre-crosslinked with 60 mM of either CaCO_3_, CaCl_2_ or CaSO_4_ at the pre-determined optimum cross-linking ratios. Using the 3D bioplotter constructs of Ø 16 mm by 2.5 mm high were printed. The printed constructs were further crosslinked post-printing by placing them in a bath of 60 mM CaCl_2_ for 1 minute.

To investigate if varying the crosslinking ratio would influence the mechanical properties of printed constructs, a 3.5% (w/v) bioink of high MW alginate was prepared in high glucose DMEM. This alginate solution was pre-crosslinked with 60 mM CaSO_4_ and the bioinks were prepared at varying crosslinking ratios (6:1, 49:10, 19:5, and 25:9). Cylinders of Ø 16 mm by 2.5 mm high were printed to preform mechanical testing. The printed constructs were further crosslinked post-printing by placing them in a bath of 60 mM CaCl_2_ for 1 minute.

All mechanical tests were performed using a single column Zwick (Zwick, Roell, Germany) with a 5 N load cell as previously described^59^, see Fig. 6(C). Briefly, stress relaxation tests were performed using impermeable metal platens, applying a 10% unconfined compressive strain with a ramp displacement of 0.001 mm/s. The Young’s modulus was defined as the slope of the linear phase of the resulting stress-strain curve during the ramp phase of the compression, while the equilibrium modulus was defined as the stress after the relaxation phase divided by the applied compressive strain.

### Cell Isolation and Expansion

Bone marrow derived mesenchymal stem cells (MSCs) were obtained from the femur of a purchased 4-month old porcine donor from an abattoir (Perma Pig) and carried out in accordance with all relevant guidelines and regulations. The bone marrow was removed from the femoral shaft and washed in growth medium, consisting of high-glucose DMEM (Biosciences, Ireland) supplemented with 10% fetal bovine serum (FBS, GIBCO, Biosciences, Ireland), 2% penicillin (100 U ml−1) streptomycin (100 μg ml−1), (Biosciences, Ireland). A homogenous suspension was achieved by triturating with a needle. The solution was centrifuged twice at 650 g for 5 min, each time the supernatant was removed. The cell pellet was triturated and the cell suspension was filtered through a 40 μm cell sieve. Following colony formation, cells were trypsinised, counted and re-plated for a further passage at a density of 5 × 10^3^/cm^2^. All expansion was conducted in normoxic conditions, expanded in growth medium where the media was changed twice weekly. Cells were used at the end of passage 3.

### Live/Dead Cell Assay

Alginate solutions of 4.4% (w/v) were prepared by dissolving the high and low MW alginate powders in sterile PBS or growth medium, as described above. Each bioink was prepared to have a seeding density of 20 × 10^6^ cells/mL. The bioink/cell suspension was prepared by resuspending the MSCs in a volume of either PBS or growth medium, depending on the experimental group, to generate a final alginate solution concentration of 3.5% (w/v). These cellular/alginate solutions were homogenously mixed using a dual syringe approach. This final cellular/alginate solution was pre-crosslinked with 60 mM of either CaCO_3_, CaCl_2_ or CaSO_4_ at the pre-determined optimum cross-linking ratios, using the dual syringe approach again. The bioinks were then cast in an agarose block to generate constructs of Ø 6 mm by 6 mm high. These constructs were crosslinked post casting by placing them in a bath of 60 mM of CaCl_2_ solution for 1 minute, to further replicate the post-printing process. Constructs were cultured in growth medium for 24 hours at normoxic conditions, see Fig. 6(D). Cell viability was established using a live/dead assay kit (Invitrogen, Bioscience). Cell-laden constructs were rinsed in sterile PBS and incubated for 1 h in Phenol free DMEM (Sigma Aldrich) containing 2 μM calcein and 4 μM of ethidium homodimer-1 (Invitrogen). After incubation, the constructs were rinsed again in PBS and imaged with Olympus FV-1000 Point-Scanning Confocal Microscope at 488 and 543 nm channels. Cell viability was quantified using Image-J software.

### Degradation Rates of the Alginates

To determine the degradation rates over time of the two molecular weight alginates, 3.5% (w/v) alginate solutions of high and low MW were prepared in high glucose DMEM. These alginate solutions were pre-crosslinked with 60 mM of either CaCO_3_, CaCl_2_ or CaSO_4_ at the pre-determined optimum cross-linking ratios, and cast in an agarose block to generate constructs of Ø 6 mm by 6 mm high. Each construct was crosslinked again post-casting in a bath 60 mM CaCl_2_ for 1 minute. Each construct was cultured in growth medium for 21 days in normoxic conditions. Media from each sample was changed twice weekly. At each time point (Day 0, 7, 14 and 21) samples were washed and snap frozen at −80 °C. Samples were lyophilised by placing the samples in a freeze dryer (FreeZone Triad, Labconco, KC, USA). Briefly, the samples were initially frozen to −30 °C at a rate of −1 °C/min, this temperature was held for 1 hour before rising to −10 °C at a rate of +1 °C/min. Once at −10 °C, this temperature was maintained under a vacuum of 0.200 mBar for 24 hours before the temperature was increased to +20 °C at a rate of +1 °C/min^60^. Each sample was then weighed using an analytical balance (Mettler Toledo, XS205).

### Protein Retention and Release

To establish if the molecular weight of the alginate influences protein release, two 3.5% (w/v) alginate solutions of high and low MW were prepared in high glucose DMEM. Two combinations of high and low MW alginate gels were also prepared in a ratio of 75:25, as previously described^49^. Using a dual-syringe approach 100ng/mL of Human Vascular Endothelial Cell Growth Factor (VEGF, Gibco Life Technologies, Gaithersburg, MD, USA) was added to the alginate solutions. Once the VEGF is mixed thoroughly the alginate solutions were pre-crosslinked with 60 mM CaSO_4_ at the pre-determined optimum cross-linking ratios for high and low MW alginate, and printed in an agarose mould to generate constructs of Ø 6 mm by 6 mm high. Each construct was cultured in growth medium for 7 days in normoxic conditions. Media from each sample was changed twice weekly. At each time point (Day 0, 3, 5, and 7) media samples were taken and snap frozen at −80 °C. Samples Printed hydrogels were also taken at Day 0 and Day 7 and snap frozen at −80 °C. An enzyme-linked immunosorbent assay (ELISA; R&D Systems) was used to quantify the levels of VEGF released by the alginates. The alginate samples were depolymerised with 1 mL of citrate buffer (150 mM Sodium Chloride, 55 mM Sodium Citrate and 20 mM EDTA in H_2_O) for 15 minutes at 37 °C. The cell culture media and depolymerised alginate samples were analysed at the specific time points detailed above. Assays were carried out per the manufacturer’s protocol (R&D Systems) and analysed on a microplate reader at a wavelength of 450 nm.

### MSC Differentiation

To establish if tuning the bioinks mechanical properties would lead to spatially directing MSC differentiation a 4.4% (w/v) alginate solution was prepared by dissolving high MW alginate in growth medium. As described in section 4.5, porcine MSCs were encapsulated in the alginate solution, at a seeding density of 20 × 10^6^, to create a final concentration of 3.5% (w/v). Two bioinks were prepared by crosslinking the alginate solution with 60 mM CaSO_4_ at two different crosslinking ratios, a soft bioink (crosslinking ratio of 6:1) and a stiff bioink (crosslinking ratio of 25:9). Constructs were printed to create three experimental groups: (1) *Homogenous Soft:* where the entire construct (Ø = 20 mm) is printed with the soft bioink; (2) *Homogenous Stiff:* where the entire construct (Ø = 20 mm) is printed with the stiff bioink; and (3) *Gradient construct:* where the core (inner Ø = 10 mm) of the construct is printed with the soft bioink and the annulus (outer Ø = 20 mm) is printed with the stiff bioink, see Fig. 6(E). Post-printing constructs were crosslinked again in a bath of 60 mM CaCl_2_ for 1 minute. Following printing the experimental groups were treated in one of two ways: (1) cultured in Osteo:Adipo medium (as described below) for 7 days or (2) taken for mechanical testing both directly after printing. The constructs that were treated to *in vitro* culture were cultured for 3 days in a 50:50 Osteogenic:Adipogenic Induction Medium, consisting of growth medium plus dexamethasone (100 nM), ascorbic acid (50 µg/mL), β-glycerolphosphate (10 mM), isobutyl-1-methylxanthine (0.5 mM), indomethacin (200 µM), and insulin (10 µg/mL). After 3 days, medium was changed to a 50:50 Osteogenic:Adipogenic Maintenance Medium, which consisted of growth medium plus dexamethasone (100 nM), ascorbic acid (50 µg/mL), β-glycerolphosphate (10 mM), and insulin (10 µg/mL, all Sigma Aldrich) for 24 hours. This cycle was repeated for a 7-day cell culture period.

### Histological Evaluation

After the 7 days of *in vitro* culture constructs were fixed with 4% paraformaldehyde solution prepared in serum free, phenol free DMEM with 0.1% (w/v) sodium azide (Sigma Aldrich), 0.1% (v/v) Triton-X-100 and 0.1% (v/v) Tween-20, for 30 minutes. The constructs were then washed with 0.1% (v/v) Tween-20 solution and using a 10-mm biopsy the core was separated from the annulus of the constructs. Each section was equilibrated with alkaline staining buffer (pH 8.2) consisting of CaCl_2_ (100 mM), Tris-Hydrochloric Acid (100 mM), 0.1% (v/v) Tween-20 and Magnesium Chloride (50 mM). In the same staining buffer constructs were incubated in 500 µg/mL Naphthol AS-MX phosphate and 500 µg/mL of Fast Blue BB (All Sigma Aldrich) for 60 minutes. Constructs were then washed again with alkaline staining buffer before being equilibrated back to neutral using DMEM containing CaCl_2_ (100 mM) and 0.1% (w/v) Tween-20. Constructs were then stained with 0.5% Oil Red O in methanol solution for 90 minutes at room temperature. Samples were fixed again in 4% paraformaldehyde solution, as described above, for 30 minutes.

### Statistical Analysis

Results are expressed as mean ± standard deviation. Young’s Modulus and Equilibrium Modulus analysis for varying crosslinking ratios was examined using one-way analyses of variance (ANOVA) with the addition of Tukey’s correction for multiple comparisons testing. All other quantitative analyses were examined using two-way ANOVA with the addition of Tukey’s correction for multiple comparisons testing. All analyses were performed using GraphPad (GraphPad Software, La Jolla California USA, www.graphpad.com). For all comparisons, the level of significance was p ≤ 0.05.

## Electronic supplementary material

Supplementary Figure 1
