# Supplementary material for: Tuning Alginate Bioink Stiffness and Composition for Controlled Growth Factor Delivery and to Spatially Direct MSC Fate within Bioprinted Tissues
Source: Sci Rep. 2017 Dec 6;7:17042. doi: 10.1038/s41598-017-17286-1 (PMC5719090; doi:10.1038/s41598-017-17286-1)
Supplement: Supplementary file 1 — Supplementary Figure 1 [file 41598_2017_17286_MOESM1_ESM.pdf]

Tuning Alginate Bioink Stiffness and Composition for Controlled Growth Factor Delivery and to Spatially Direct MSC Fate within Bioprinted Tissues

Fiona E Freeman[1,2], Daniel J Kelly[1,2,3,4]*

[1]Trinity Centre for Bioengineering, Trinity Biomedical Sciences Institute, Trinity College Dublin, Ireland.

[2]Department of Mechanical and Manufacturing Engineering, School of Engineering, Trinity College Dublin, Ireland.

[3]Department of Anatomy, Royal College of Surgeons in Ireland, Dublin, Ireland.

[4]Advanced Materials and Bioengineering Research Centre (AMBER), Royal College of Surgeons in Ireland and Trinity College Dublin, Dublin, Ireland.

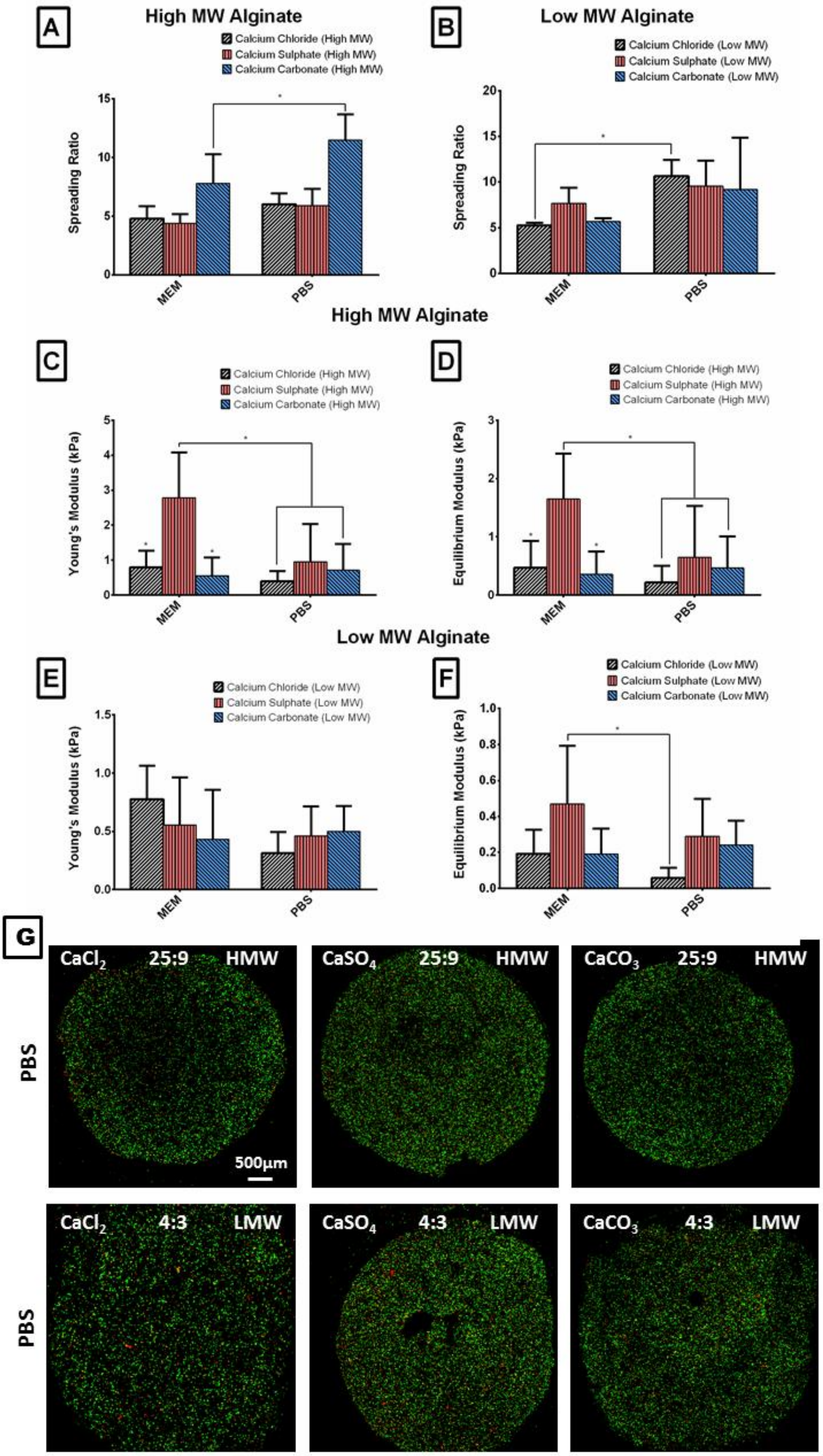
A
High MW Alginate
Calcium Chloride (High MW)
Calcium Sulphate (High MW)
Calcium Carbonate (High MW)
Spreading Ratio
MEM
PBS
B
Low MW Alginate
Calcium Chloride (Low MW)
Calcium Sulphate (Low MW)
Calcium Carbonate (Low MW)
Spreading Ratio
MEM
PBS
High MW Alginate
C
Young's Modulus (kPa)
D
Equilibrium Modulus (kPa)
Low MW Alginate
E
Young's Modulus (kPa)
F
Equilibrium Modulus (kPa)
G
CaCl2 25:9 HMW
CaSO4 25:9 HMW
CaCO3 25:9 HMW
PBS
500μm
CaCl2 4:3 LMW
CaSO4 4:3 LMW
CaCO3 4:3 LMW
PBS

Supplementary Figure 1: Effects of preparing the bioinks in PBS. Spreading ratio for **(A)** high and **(B)** low MW Alginate prepared in MEM vs. PBS. *$p<0.05$ vs same group prepared in MEM. **(C)** Young's Modulus and **(D)** Equilibrium Modulus for the high MW alginate prepared in MEM vs. PBS. *$p < 0.05$ vs. $CaSO_4$ crosslinked bioink prepared in MEM. **(E)** Young's Modulus and **(F)** Equilibrium Modulus for the low MW alginate prepared in MEM vs. PBS. *$p < 0.05$ vs. $CaSO_4$ crosslinked bioink prepared in MEM. **(G)** Representative images of Live/Dead staining used to determine cell viability of $CaCl_2$, $CaSO_4$ and $CaCO_3$ crosslinkers for both high (HMW) and low MW (LMW) alginate at the previously determined optimum crosslinking ratio (25:9 and 4:3 respectively). All bioinks were prepared in PBS. Error bars denote standard deviation, n = 6.
